# Genomics-informed outbreak investigations of SARS-CoV-2 using civet

**DOI:** 10.1371/journal.pgph.0000704

**Published:** 2022-12-09

**Authors:** Áine O’Toole, Verity Hill, Ben Jackson, Rebecca Dewar, Nikita Sahadeo, Rachel Colquhoun, Stefan Rooke, J. T. McCrone, Kate Duggan, Martin P. McHugh, Samuel M. Nicholls, Radoslaw Poplawski, The COVID-19 Genomics UK (COG-UK) Consortium, COVID-19 Impact Project (Trinidad & Tobago Group), David Aanensen, Matt Holden, Tom Connor, Nick Loman, Ian Goodfellow, Christine V. F. Carrington, Kate Templeton, Andrew Rambaut

**Affiliations:** 1 Institute of Evolutionary Biology, University of Edinburgh, Edinburgh, United Kingdom; 2 Department of Clinical Microbiology, NHS Lothian, Edinburgh, United Kingdom; 3 Department of Preclinical Sciences, The University of the West Indies, St. Augustine, Trinidad & Tobago; 4 Public Health Scotland, Glasgow, United Kingdom; 5 School of Medicine, University of St Andrews, St Andrews, United Kingdom; 6 Institute of Microbiology and Infection, University of Birmingham, Birmingham, United Kingdom; 7 The Centre for Genomic Pathogen Surveillance, Big Data Institute, University of Oxford, Oxford, United Kingdom; 8 Pathogen Genomics Unit, Public Health Wales NHS Trust, Cardiff, United Kingdom; 9 School of Biosciences, The Sir Martin Evans Building, Cardiff University, Cardiff, United Kingdom; 10 Quadram Institute, Norwich, United Kingdom; 11 Department of Pathology, University of Cambridge, Cambridge, United Kingdom; University of Sao Paulo: Universidade de Sao Paulo, BRAZIL

## Abstract

The scale of data produced during the SARS-CoV-2 pandemic has been unprecedented, with more than 13 million sequences shared publicly at the time of writing. This wealth of sequence data provides important context for interpreting local outbreaks. However, placing sequences of interest into national and international context is difficult given the size of the global dataset. Often outbreak investigations and genomic surveillance efforts require running similar analyses again and again on the latest dataset and producing reports. We developed civet (cluster investigation and virus epidemiology tool) to aid these routine analyses and facilitate virus outbreak investigation and surveillance. Civet can place sequences of interest in the local context of background diversity, resolving the query into different ’catchments’ and presenting the phylogenetic results alongside metadata in an interactive, distributable report. Civet can be used on a fine scale for clinical outbreak investigation, for local surveillance and cluster discovery, and to routinely summarise the virus diversity circulating on a national level. Civet reports have helped researchers and public health bodies feedback genomic information in the appropriate context within a timeframe that is useful for public health.

## Introduction

The timely sharing of genomic data during the SARS-CoV-2 pandemic has enabled large-scale national and international surveillance efforts around the world. On a finer scale, pathogen genomics can supplement infection prevention and control efforts in clinical settings, as well as aid in outbreak investigations in community settings [1–4]. However, the intense SARS-CoV-2 sequencing effort has produced a genomic dataset orders of magnitude larger than any previous epidemic, with more than 13 million sequences shared publicly at time of writing. It is therefore challenging to effectively condense information into relevant summaries and provide meaningful context in a timeframe that allows the data to be of immediate use to those involved in local outbreak response.

Analysing or interpreting genomic information alone without relevant epidemiological information can be misleading and lead to incorrect conclusions due to the incomplete nature of the data. The relatively low mutation rate of SARS-CoV-2, frequent occurrence of convergent mutations (homoplasies), and prevalence of incomplete genome sequences make it critical to integrate epidemiological information alongside the genomic data to provide the most accurate picture and extract the most value from any given dataset. This includes temporal and spatial information, but may also include outbreak-specific data such as profession, ward, clinical metadata, or the background of viral lineages actively circulating in the community. Outbreak investigations often require bespoke reports that present information in a transparent and accessible manner. The data presented must be easily interpretable by health care providers and teams involved in infection control, the majority of whom are not accustomed to incorporating this type of data into their decision making processes.

The virus genomics community has developed a number of tools for analysing and visualising virus genomic data on the order of magnitude of this pandemic. HgPhyloPlace uses UShER to rapidly place sequences of interest into a global SARS-CoV-2 phylogeny (https://hgwdev.gi.ucsc.edu/cgi-bin/hgPhyloPlace) [5]. Tree visualization tools such as Pando (pando.tools), cov2tree (cov2tree.org) [6], Microreact [7] and Dendroscope [8] can efficiently display phylogenies with a million sequences, and tools like ClusterTracker can estimate and summarise geographic introductions [9]. However, even with these innovations, it is challenging to construct a phylogenetic tree of that size, given the particular challenges of SARS-CoV-2 data [10, 11]. Furthermore, this approach is reliant on volunteers maintaining a global SARS-CoV-2 phylogeny and future epidemics or pandemics may not have such a resource available. NextStrain takes an alternative approach and downsamples the dataset heavily, leaving a manageable amount of data to display [12]. The advantage is a rapidly generated phylogeny, however only a small subset of the full diversity is represented. Approaches to condense SARS-CoV-2 genomic information by Single Nucleotide Polymorphism (SNP) typing or lineage typing–such as scorpio (github.com/cov-lineages/scorpio), aln2type (github.com/connor-lab/aln2type) and pangolin [13]–have been useful but present one dimensional data.

We developed civet (Cluster Investigation and Virus Epidemiology Tool) to address this challenge of integrating metadata while condensing huge quantities of genomic data, and thereby aid SARS-CoV-2 outbreak investigations and surveillance efforts. Civet enables robust phylogenetic analysis to be performed, dynamically querying a large background dataset and generating interactive reports integrating both epidemiological metadata and genomic analysis. Both Public Health Scotland and Public Health England have routinely used civet to inform local outbreaks of SARS-CoV-2 and a number of studies have already been published that have used civet as tool for genomic epidemiology [14–17].

## Methods

Civet is a Python-based tool with an embedded analysis pipeline implemented in Snakemake [18]. Civet outputs the analysis as a customisable, interactive HTML report. We developed civet as part of the ARTIC Network (artic.network) and COVID-19 Genomics UK (COG-UK) [19] projects and it has been hosted on CLIMB-COVID [20], an isolated partition of the Cloud Infrastructure for Microbial Bioinformatics (CLIMB), since July 2020 [21]. Public health agencies and researchers across the UK use civet routinely to aid SARS-CoV-2 outbreak investigations and generate local surveillance reports.

### Ethics statement

All the scenarios presented as example cases for civet use are synthetic examples inspired by actual data and details have been entirely anonymised and/or altered. The first case study is a constructed outbreak loosely based on a hospital outbreak, but details have been masked. The second case study uses only publically available data from COG-UK. The UK studies were done as part of surveillance for COVID-19 infections under the auspices of Section 251 of the National Health Service Act 2006 or Regulation 3 of The Health Service (Control of Patient Information) Regulations 2002, or both. They therefore did not require individual patient consent or ethical approval. The COVID-19 Genomics UK Consortium study protocol was approved by the Public Health England Research Ethics Governance Group (reference number R&D NR0195). The third case study in Trinidad and Tobago is wholly based on public data.

### Background data

**Fig 1:**
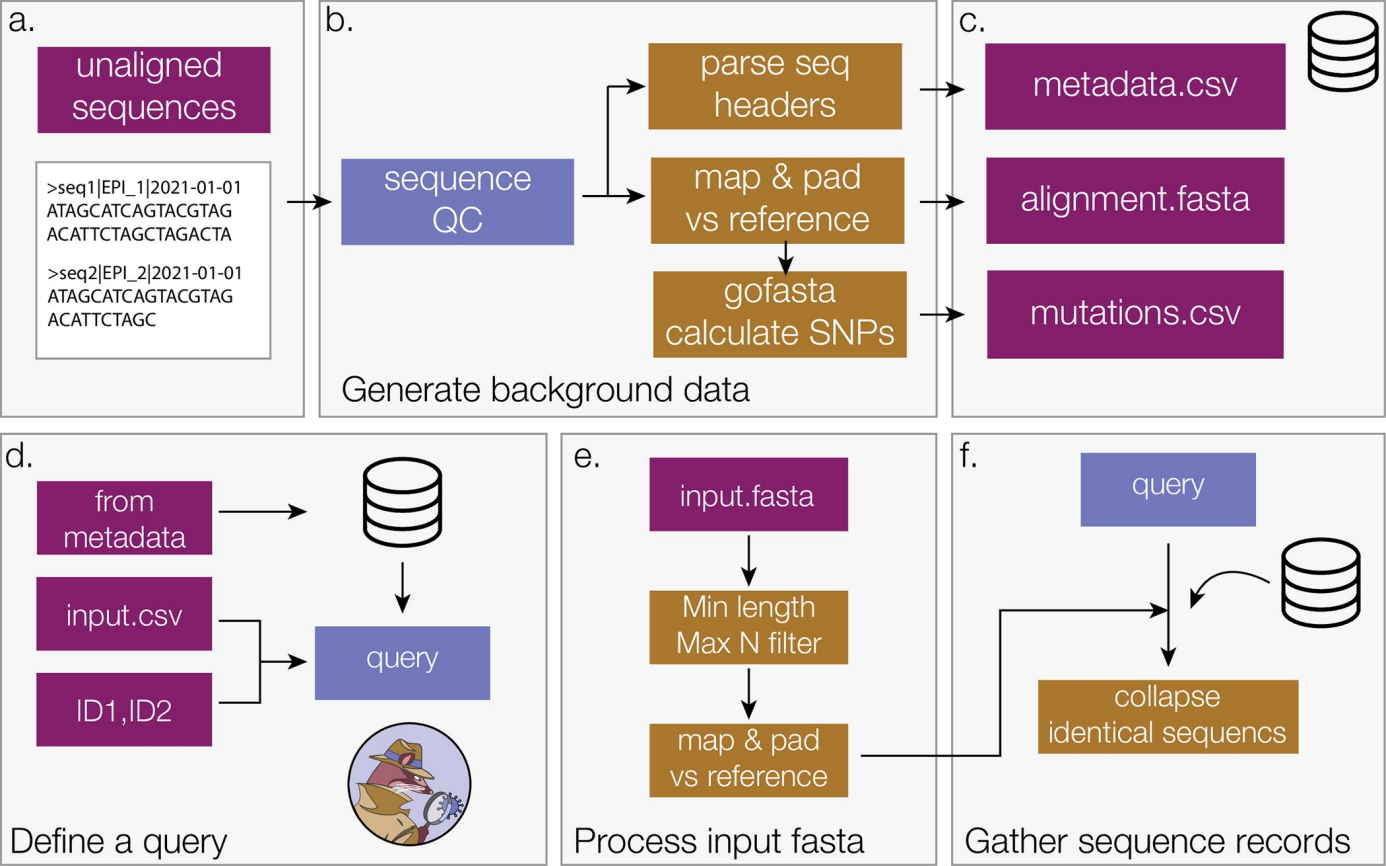
Background data generation pipeline (a-c) and how a civet query is defined (d-f). In order to contextualize the query sequences, civet requires a set of background data files, minimally an alignment and metadata file. a) These files can be generated from an unaligned multi-sequence file using the flag:—generate-background-data parse_seq_headers. Alternatively, the latest downsampled Augur download hosted on GISAID can be converted into civet background data using the flag:—generate-background-data align_curate. b) The genome sequences are put through a minimum length and maximum N-content filter before being mapped against a reference sequence. The alignment file is generated by trimming the genome sequences to the protein coding region (positions 265 to 29674) defined by the SARS-CoV-2 reference genome (Genbank ID: NC_045512.2), masking the untranslated regions (UTRs) with Ns. The SARS-CoV-2 reference genome and coordinates are used by default, however this can be configured for a different genome and different coordinates. Information encoded in the sequence header is used to generate the metadata file. gofasta condenses the alignment to the set of derived nucleotide changes in each sequence with respect to the root of the pandemic, to provide an extra speed up for analysis within civet. c) The background files created can then be used as the background data for civet with—datadir or set as an environment variable. d) The query is generated from the background data supplied by specifying a set of criteria to match against, for example all sequences from a particular location within a certain timeframe. The user can also provide a string of specific ids to match or an additional metadata file that specifies the query records and may contain extra metadata fields that only correspond to query sequences, for example patient IDs. e) An additional fasta file for sequences not present in the background data can be provided and civet will perform some quality control checks and align the sequences by mapping and padding against the reference (Default NC_045512.2). f) civet combines the set of query sequence records matched from the background data and from the input fasta file to generate the full query set, and then collapses identical sequences for efficiency. These get expanded out at the end of the analysis pipeline.

To run civet, the user must minimally provide a sequence alignment and metadata file representing the background diversity of the pathogen of interest. Users on CLIMB-COVID have this data provided by the COG-UK Datapipe (https://github.com/COG-UK/datapipe) although a similarly centralised set up could be applied elsewhere. The COG-UK Datapipe filters the input data to only include sequences of high quality with more than 90% genome completeness and removes any non-UK outliers with a genetic distance from the root beyond four standard deviations from the mean of the dataset in each epiweek. The sequences are aligned against a reference genome sequence (the canonical SARS-CoV-2 reference genome Genbank ID: NC_045512.2) and problematic sites such as the untranslated regions (UTRs) are masked out. Any background dataset used with civet should strive to be of the highest quality possible as catchment finding and phylogenetic inference will be sensitive to poor quality data. Civet can generate the background alignment, metadata file and a SNP summary file from an unaligned fasta sequence file, such as a download sequence file from GISAID [22] with metadata embedded in the header with the pipeline parse_seq_headers (Fig 1). This short pipeline first filters genome sequences based on a minimum length and maximum ambiguity content (%N) cut-off. It then maps against a reference sequence (default is the SARS-CoV-2 reference genome Genbank ID: NC_045512.2, but any reference genome can be supplied) using minimap2 v2.17 [23]. The resulting sam file is converted to fasta format with the 5’ and 3’ untranslated regions (UTRs) masked using gofasta [24]. We generate the background metadata file by parsing information from the sequence headers. Civet also has a curation pipeline (align-curate) that can take the latest downsampled SARS-CoV-2 dataset generated by the Augur pipeline [25] hosted on GISAID and convert it into the format required for civet. A full step-by-step guide on accessing and generating this background dataset can be found at cov-lineages.org/resources/civet.

### Input options

There are two main ways to define a query dataset, described in Fig 1D. First, a user can define a query from the background data based on metadata, for instance a collection date within a certain time frame, or sequences from a particular location. For example, to generate a report for sequences from June 2021 sampled in Edinburgh: civet—from-metadata date = 2021-06-01:2021-07-01 location = Edinburgh. Alternatively, the user can supply a string of query identifiers directly to civet, or a comma-separated (CSV) file specifying the query sequences with some additional metadata not present in the background, like patient IDs. Optionally, a separate fasta file can be supplied to run an analysis on sequences not present in the background dataset. The sequences will go through configurable quality control filters for minimum sequence length and maximum N-content, and are then aligned by mapping against the reference sequence and padding with Ns as described for the background dataset creation (Fig 1E).

### Handling of insertions and deletions within the analysis pipeline

This approach of aligning to reference is standard across many of the analytical pipelines available for SARS-CoV-2. In this way alignment errors are minimised and alignment can be performed within a timeframe that enables analysis of such large datasets as are available for SARS-CoV-2. A limitation of this approach is that it consumes any insertion mutations that might have occurred. Insertions are relatively rare and this is a time-saving alignment approximation that has become standard for SARS-CoV-2. Deletions are represented in the alignment produced within civet. One of the flags within civet (—mutations) allows the user to specify mutations of interest and this can include deletions as well as SNPs. For catchment finding, deletions are ignored in a pairwise manner, as is common practice in many phylogenetic methods. Similarly, within iqtree deletions are classified as unknown and so do not influence the tree structure in the final report.

### Analysis pipeline

**Fig 2:**
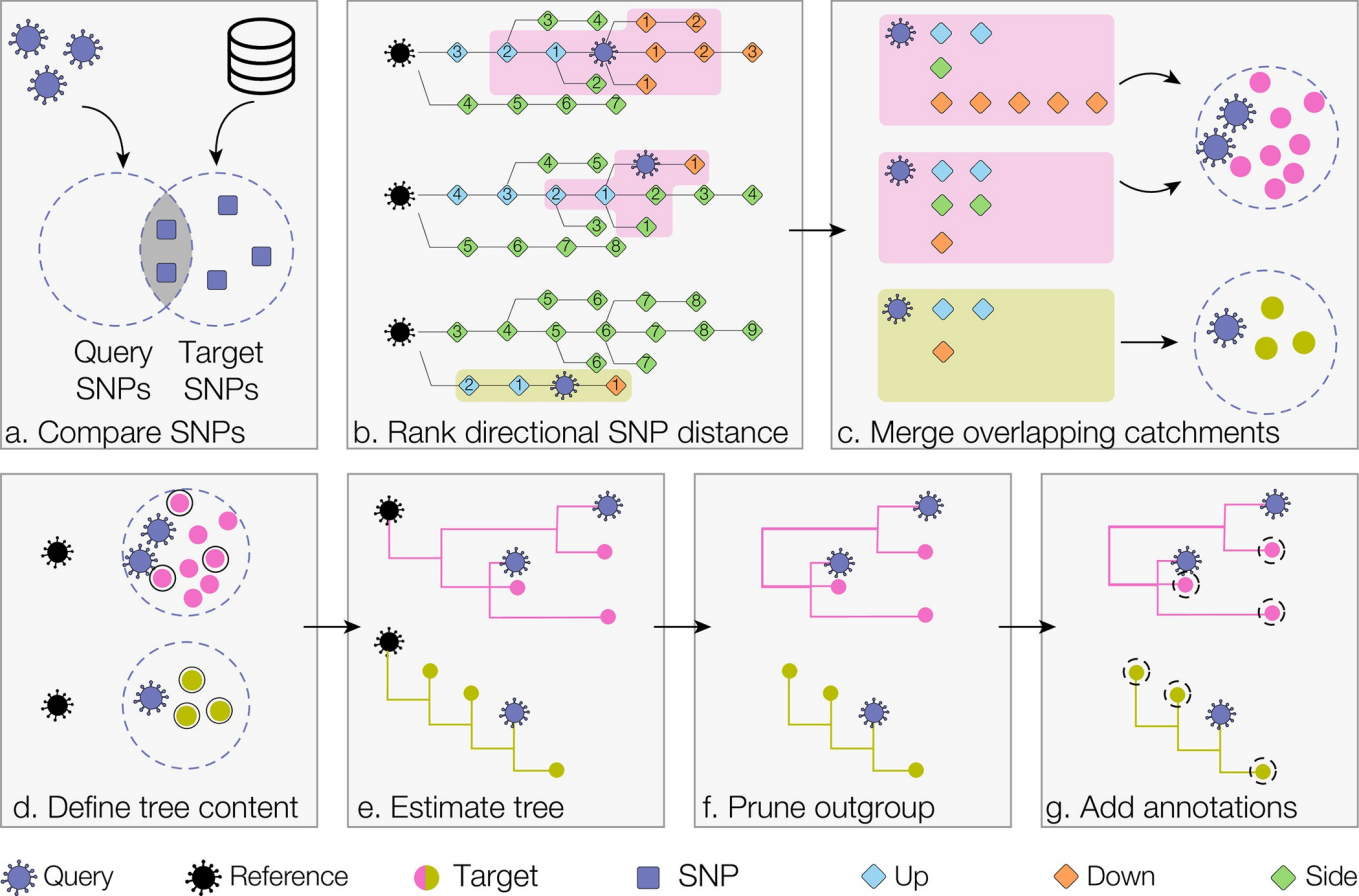
Schema of civet catchment and tree building pipeline. We show three query sequences, falling in two distinct catchments (pink and green). a) Each query sequence is compared against the set of SNPs for every record (target) in the background metadata. By evaluating the intersection and union of the two SNP sets, it is possible to assess directional SNP distance relative to the reference sequence (the early lineage A sequence with GISAID ID EPI_ISL_406801). b) For each query, all targets are ranked by distance from the query and classified as either up, down or side targets based on the set profile in panel a. c) Catchments are constructed by selecting all targets that fall within the specified SNP distance. Up, down and side distances can be configured separately (the default SNP distance of 2 SNPs for all categories is shown here). Civet then merges any catchments with overlapping targets. d) An outgroup reference sequence is added to each catchment and, if necessary, catchments are downsampled. e) Civet estimates a maximum likelihood tree for each catchment using iqtree. f) The reference sequence is pruned out and the tips of the tree are annotated with user-specified fields. g) Specific metadata annotations are added to each tip, which can be toggled within the report.

Query identifiers are matched with the alignment in the background data, and the set of fasta sequence records is compiled from queries in both alignment files. Identical sequences are collapsed to a single unique sequence (Fig 1F). Collapsing identical sequences greatly improves analysis efficiency, particularly for outbreak investigations of epidemiologically linked sequences. Once identical sequences have been collapsed, civet searches the background dataset using the ‘updown topranking’ method in gofasta v0.0.5. [24] to identify the local set of sequences most similar to each query. Comparing the set of derived SNPs in each query with the set of derived SNPs in every record (target) in the background dataset (Fig 2A) this algorithm can efficiently extract genetically similar genomes from a dataset comprising millions of records. As illustrated in Fig 2A, SNPs can either be unique to the query sequence, unique to the target sequence, or present in the intersection of the two. SNPs present in the intersection represent shared ancestry whereas an excess of SNPs in either the query or target set can be interpreted to give directionality relative to a root sequence. These set comparisons (details in S1 Fig) allow the target sequences to be classified as either on a polytomy with (same), a direct ancestor of (up), a direct descendant of (down) or polyphyletic with (side) the query sequence (Fig 2). Each target is then ranked according to SNP distance from the query sequence (as illustrated in the schema in Fig 2B). The customisable SNP distance is used to define which target sequences fall within the catchment of a given query. All equally distant targets are included in the catchment. For a given query, if no targets fall within the SNP distance cut off, the algorithm continues outwards in all directions and attempts to get at least one sequence per category (up, down or side). This results in a set of targets for each query, and any queries with overlapping targets have their catchments merged together (Fig 2C).

The gofasta method updown topranking has parameters that can deal with some missing data. By default and within civet, any sites that are different between the reference genome and the query genome are catalogued and for every pairwise comparison there is 10% ambiguity at these sites allowed in total. Within civet there is also a maximum ambiguity allowed for a query sequence to be processed and a catchment found (default is 50% ambiguity. By using correct Pango lineage assignment (as defined in Rambaut et al. 2020 [26]) as a proxy for the appropriate catchment, we ran simulations that assessed the extent to which ambiguities in the query sequence impacted the catchment content for a given query sequence (S2 Fig). We observe that this method is sensitive to missing SNPs so to ensure the most accurate results we recommend using only high-quality data as part of the background and query dataset. As part of CLIMB-COVID, the COG-UK datapipe only allows sequences of greater than 90% genome completeness through to the civet background data and we recommend applying a similar threshold in a custom database. Additionally, users may wish to independently validate the catchment for particularly low-quality sequences using the hgPhyloPlace tool which uses parsimony to place sequences within a global SARS-CoV-2 tree maintained by the UShER team (genome.ucsc.edu/cgi-bin/hgPhyloPlace).

At this point in the pipeline, there is no limit to the size of catchments and as the pandemic has been sampled so intensively in some areas, even relatively low SNP distances can lead to a large catchment. The user has the option to downsample the catchments prior to tree building and configure the maximum number of the background sequences to include in a given catchment tree (Fig 2D). Downsampling can be run in: random mode, which randomly samples from the full catchment; enrich mode, which allows the user to specify a metadata trait to enrich for and the factor by which to enrich over the other targets in the catchment; or normalise mode, which allows the user to sample evenly across a metadata trait, such as epiweek. The query sequences, background catchment sequences and an anonymised early lineage A outgroup sequence are then gathered for tree building. Each catchment tree along with the queries is then estimated using iqtree with the HKY substitution model, in fast mode [27, 28] (Fig 2E). The civet software then prunes the outgroup from the resulting maximum likelihood trees and annotates them with user-specified metadata traits (Fig 2F and 2G). Optionally, the user can search for mutations of interest and investigate which nucleotide or amino acid variant is present at sites in both the queries and background catchment sequences, and can also annotate these in the catchment trees.

### Report content

**Fig 3:**
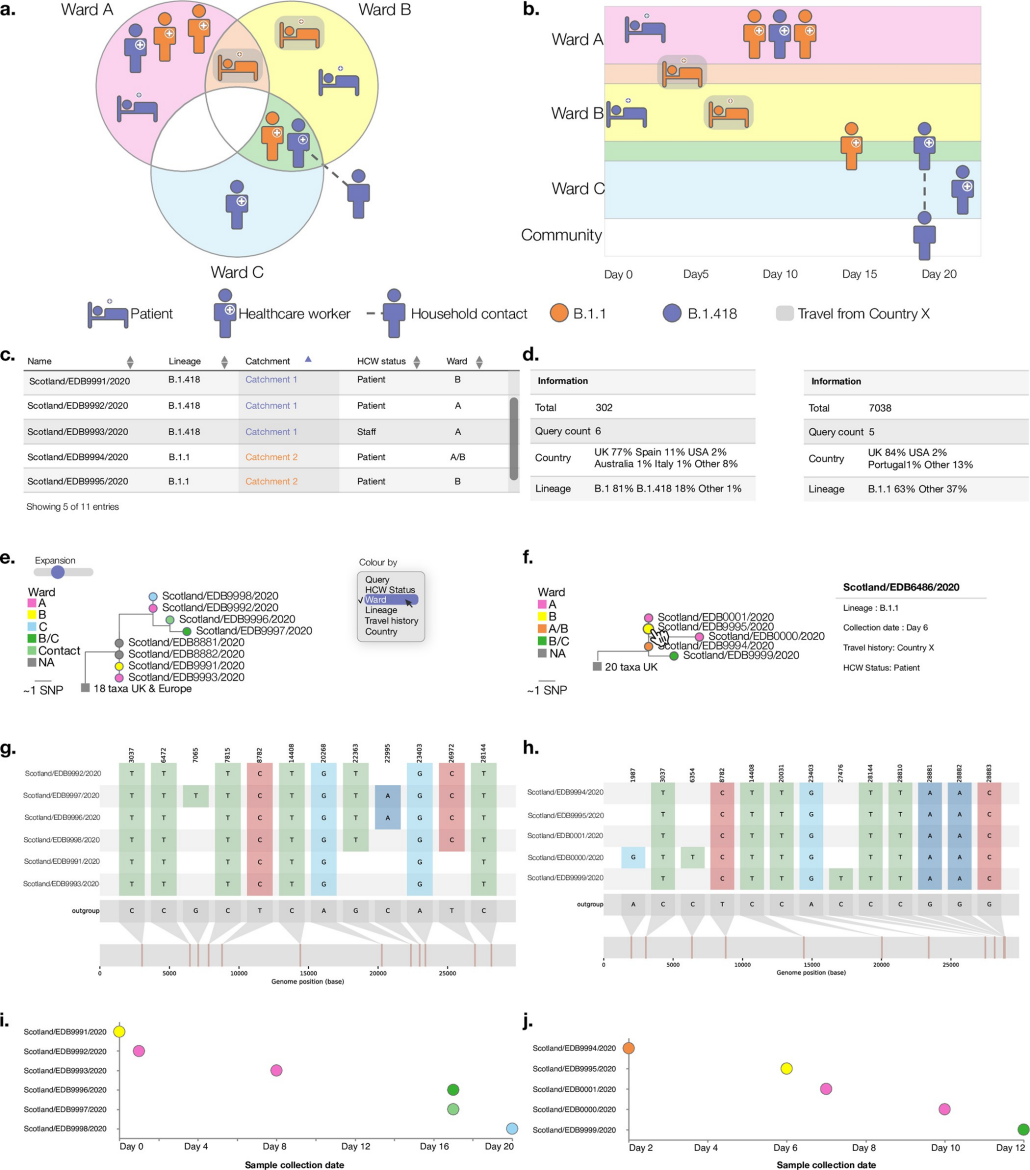
Schema of clinical outbreak investigation June 2020, colour of cases indicate lineage revealed by genome sequencing (B.1.1 or B.1.418) (a-b) and components of a civet report generated for the outbreak investigation (c-j). a) The outbreak occurred across three wards and involved six members of staff, four patients and one household contact of a staff member. b) Timeline of sample collection dates across wards A, B and C. c) The metadata of all query sequences is summarised in an interactive table, with sortable columns that can be toggled on and off. d) Each catchment is summarised in full, regardless of downsampling. Number of queries and the countries and lineages within the catchment are indicated. e) The catchment phylogenies are displayed initially in compact form, but can be expanded vertically using the Expansion slider. By default tip nodes are coloured by whether a tip is a query taxa or not, but the dropdown menu allows the user to colour tip nodes by any trait specified in—tree-annotations. f) Tip nodes can be selected to show the metadata associated with that particular sequence and clades can be collapsed to a single node by selecting the parent branch. g-h) snipit graphs highlight nucleotide differences from the reference genome. i-j) A timeline summarises any query date information provided. Note: all metadata has been de-identified for data protection purposes.

Civet generates a fully customisable report, summarising information about the queries of interest and the surrounding diversity. The report generated is a HTML file that can be viewed in a web browser, thus allowing the interactivity of web-pages. The components of the report include an interactive table summarising metadata of the query sequences, including any user supplied metadata; which catchment a query falls in; and the mutations of interest if specified. This table can be sorted, filtered and its columns can be dynamically configured, all within the distributable report. For each catchment, the civet report contains a table summarising the catchment content (prior to downsampling) and describes which lineages and countries are present in this local diversity neighbourhood (example shown in Fig 3D).

The civet report displays the catchment trees using the interactive tree visualisation library FigTree.js (https://github.com/rambaut/figtree.js). The trees can be expanded out along the vertical axis and tip nodes can be coloured by any field specified with annotations—tree-annotations. Clades can be collapsed down by clicking on the parent branch and uncollapsed by clicking again. Each taxa in the tree is associated with additional metadata that can be displayed by selecting a tip (demonstrated in Fig 3F). Civet runs snipit, a python tool that finds the SNPs relative to a reference in a multiple sequence alignment and highlights these changes as a figure (https://github.com/aineniamh/snipit). The report also contains a query timeline based on supplied temporal metadata, and interactive maps both for plotting the query sequence locations and for summarising the background diversity in the location of interest up to administrative level 2 for the UK and administrative level 1 for the rest of the world.

The user can generate multiple reports with one command to customise content for different intended audiences. Using the—report-content option, a report containing all the results shown in Fig 3 can be generated alongside a report intended for the Infection Prevention and Control (IPC) team, which may just contain the summary tables for instance and not the phylogenies Full report configuration details can be found at the civet documentation at https://cov-lineages.org/resources/civet.html.

## Results

### Case study 1: Hospital outbreak

There have been a number of studies demonstrating the utility of in-hospital genomic epidemiology for outbreak investigation to supplement standard infection prevention and control (IPC) practices e.g. [1, 2, 29]. To aid in these investigations, which generally involve standard bioinformatic and phylogenetic methods and report generation, civet can contextualize sequences of interest and generate distributable routine reports.

The case study presented in Fig 3 describes an outbreak investigation carried out in an Edinburgh hospital in 2020. An outbreak of SARS-Cov-2 was detected, with cases across three wards that included multiple staff and patients (Fig 3A). The earliest case detected was a patient in Ward B sampled on Day 0 (Fig 3B). In the following days, three more patients across Wards A and B tested positive for SARS-CoV-2, two of whom had recently travelled from Country X. Subsequently, three healthcare workers who had been working in Ward A and two healthcare workers who had been working across Wards B and C tested positive. A household contact of one of these healthcare workers tested positive the same day and finally a healthcare worker in ward C tested positive. At the outset of the investigation, the outbreak was thought to have been caused by either an initial patient to staff transmission event with subsequent staff to staff transmission, or multiple patient to staff exposures.

Genome sequencing of SARS-CoV-2 samples from staff and patients revealed the outbreak consisted of two distinct clusters, or catchments, corresponding to PANGO lineages B.1.1 and B.1.418. Fig 3C–3J summarises the content of the default report produced by civet, full report available at https://cov-lineages.org/resources/civet/civet_case_study_1.html. Fig 3C displays the interactive query summary table and catchment summary tables (Fig 3D). The phylogenies in Fig 3E and 3F are coloured by ward. Fig 3E shows the phylogenetic relationship of queries present in catchment 1, alongside the background sequences. Two community samples also from Edinburgh sit on a polytomy with, and are identical to, the earliest patient case detected in Ward B. Particularly with SARS-CoV-2 it’s not possible to infer directionality based on this information, however this phylogeny does show that the diversity in the hospital overlapped with that present in the community. Fig 3F shows the phylogenetic relationship of catchment 2, with the two patients with travel history from Country X and earliest staff member to contract lineage B.1.1 all sharing identical SARS-CoV-2 genome sequences. Fig 3G and 3H displays the snipit plots that summarise the nucleotide changes from reference among queries of interest, and the sample collection date for each query sequence is shown in the timeline plot in Fig 3I and 3J, coloured by ward. civet resolved the outbreak into two distinct catchment trees making it likely that there were multiple introductions into the hospital from the community, and the mixture of wards present in each catchment implies some between-ward transmission. In this case, the use of civet uncovered a patient to healthcare worker transmission event, indicating that better PPE may have been required for staff; as well as separate introductions into wards, implying a need for tighter restrictions on visitors or more thorough screening of incoming patients. As the case was deemed at least two separate introduction events with clear transmission links, the outbreak investigation was subsequently closed by the IPC team.

### Case study 2: Community surveillance

**Fig 4:**
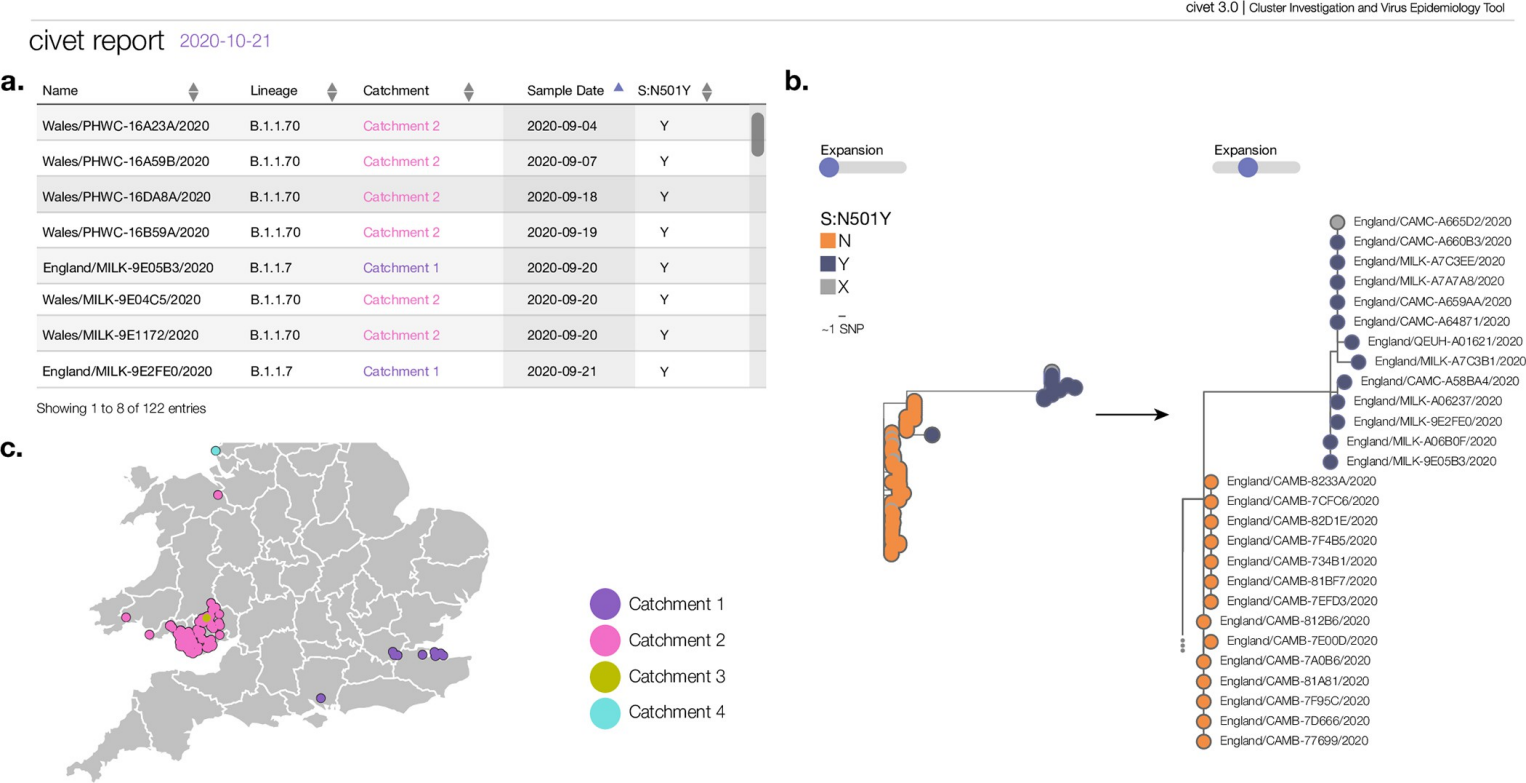
Sample of figures from a civet report demonstrating its use for community surveillance in the UK. As a hypothetical example, we used civet to search the COG-UK dataset from the 21st of October 2020 for SARS-CoV-2 sequences with the spike protein mutation N501Y in September and October 2020. At this point, 4 independent occurrences of this mutation were detected using civet. The earliest sequences can be seen in panel a. The two main clusters correspond to B.1.1.70, which was a lineage circulating in Wales, and B.1.1.7, which only had 13 sequences at this time point. Despite being small, the striking basal branch of B.1.1.7 is clearly visible in panel b. Running civet routinely enables early identification and tracking of clusters such as these. Panel c shows the query map of the samples identified with N501Y and the geographic separation of catchments 1, 2 and 4. The polygon data for the figure has been sourced from the Global Administrative Database https://geodata.ucdavis.edu/gadm/gadm4.1/shp/gadm41_GBR_shp.zip and can be used for academic use and for publication under a under an open licence such as CC-BY (https://gadm.org/license.html).

Civet can also be used as part of routine local surveillance to summarise the diversity of viruses circulating in a local area or to flag and monitor clusters of interest. The N501Y mutation in the SARS-CoV-2 spike protein has been predicted to increase SARS-CoV-2 receptor binding domain ACE2 affinity (https://jbloomlab.github.io/SARS-CoV-2-RBD_DMS/ last accessed 2021-08-10) [30]. As such, the presence of this mutation has been monitored as part of the genomic surveillance efforts in the UK and around the world. We present a hypothetical case of a civet report generated from a simple command used to search a background dataset from COG-UK from the 21st of October 2020 (Fig 4, full report available at https://cov-lineages.org/resources/civet/civet_case_study_2.html). The search defined queries as sequences from the UK with the spike N501Y mutation from the beginning of September 2020 to the latest data in the background set (2020-10-21). Fig 4A demonstrates the query summary table sorted by earliest samples. At the time in the UK, two concurrent geographically-distinct clusters existed (Fig 4C); one in Wales that became known as B.1.1.70 and one in south east England that became B.1.1.7. There were also two further, very small, clusters that contained S:N501Y between 1st September and 21st October 2020. At this snapshot in time, B.1.1.7 is clearly distinguishable but only has 13 sequences. By the time that the localised lockdowns which were put into place in England in response to the highly transmissible B.1.1.7, it had already seeded much of the country [31]. By running civet routinely, the user can both discover and monitor clusters such as B.1.1.7 and B.1.1.70 as they progress, and these measures can be brought in faster and localised interventions can be enacted in time to have a better effect.

### Case study 3: National surveillance

**Fig 5:**
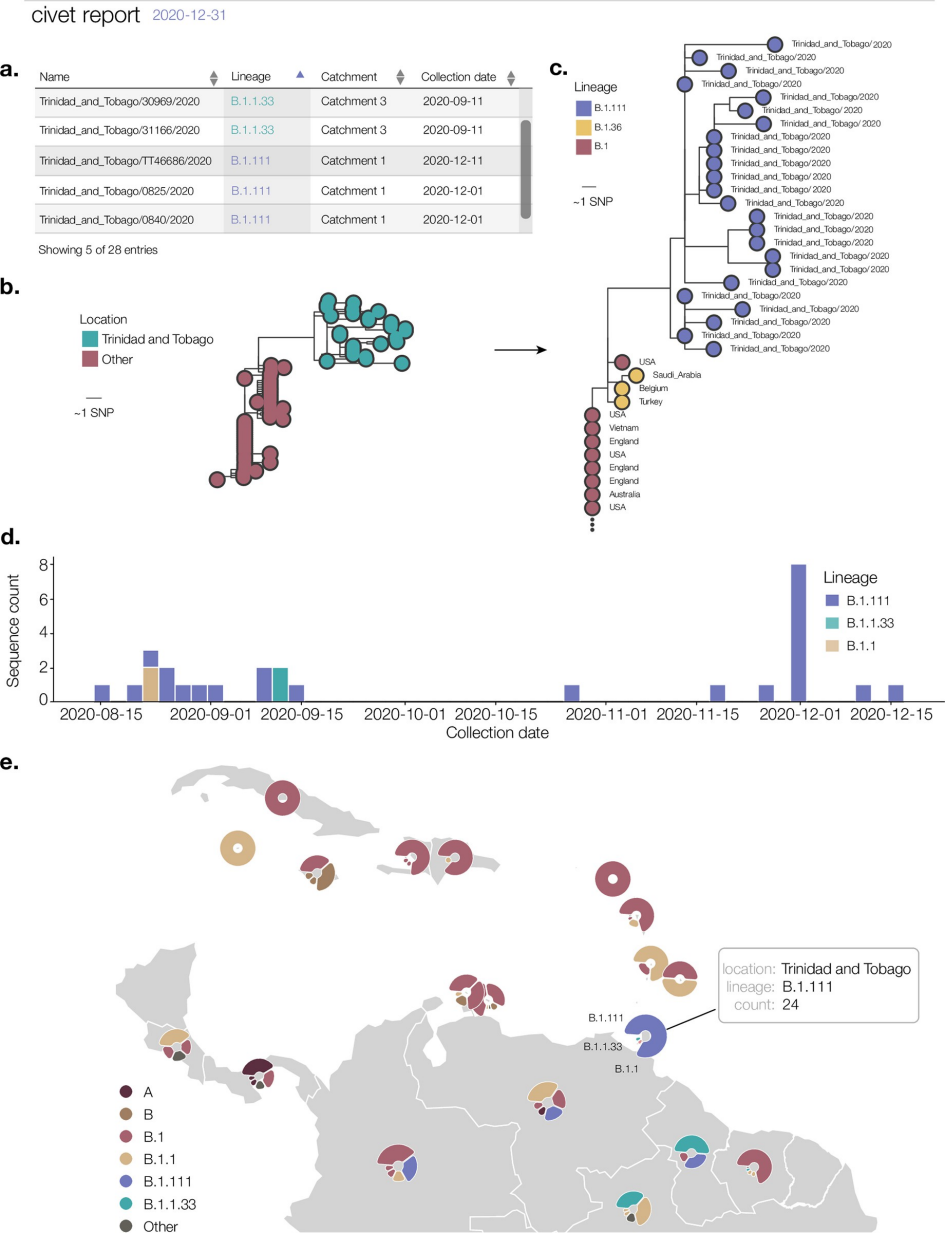
Schema of a national level surveillance report generated using civet for Trinidad and Tobago. All SARS-CoV-2 genome sequences on GISAID from 2020 with <20% ambiguity content are summarised in the report (n = 28). a. Available metadata for query sequences from Trinidad and Tobago. Most genomes have been assigned lineage B.1.111, although a smaller number of genomes are assigned other lineages B.1.1.33 and B.1.1. b. Catchment 1 phylogeny. Query sequences are placed in the context of background diversity beyond Trinidad and Tobago. c. Expanding the phylogeny and colouring tips by lineage shows this catchment includes query sequences from lineage B.1.111. d. Aggregate count of queries over time, coloured by lineage. e. Lineage diversity of Trinidad and Tobago and surrounding countries as generated using the background diversity map in civet. The base layer of the map is from Natural Earth (www.naturalearthdata.com/download/110m/physical/ne_110m_land.zip) and is in the public domain (https://www.naturalearthdata.com/about/terms-of-use/).

Civet also has the flexibility to inform surveillance efforts at the national level. In Fig 5, we show a schema of a civet report summarising genomic surveillance efforts in Trinidad and Tobago during 2020, full report available at https://cov-lineages.org/resources/civet/civet_case_study_3.html. Fig 5A displays the Trinidad and Tobago sequences alongside the available metadata, and summarises how many distinct catchments the genomes are represented by. Sequences from Trinidad and Tobago fall within three catchments, which correspond to lineages B.1.111, B.1.1 and B.1.1.33. The presence of three distinct catchments indicates there were at least three independent introductions into Trinidad and Tobago during 2020. Fig 5B and 5C show the phylogeny for catchment 1. The Trinidad and Tobago sequences form a monophyletic cluster within the background diversity of sequences from countries around the world. The timeline of events can be seen in Fig 5D, with lineage B.1.111 appearing throughout the latter half of 2020, and B.1.1 and B.1.1.33 appearing only transiently. We summarise the background diversity of other nations with SARS-CoV-2 genome data from 2020 on public databases in Fig 5E. Trinidad and Tobago is highlighted with a schema of the tooltip available in the interactive civet report. This report gives a picture of how Trinidad and Tobago fits into the overall diversity of SARS-CoV-2 in 2020. Reports could be routinely generated on a weekly or monthly basis to provide information on the changing context of a country’s epidemic compared to its neighbours. This could provide early warning on the arrival of new variants, allowing the pre-emptive organisation of non-pharmaceutical interventions such as mask mandates and the ramping up of vaccination campaigns.

## Discussion

Virus genome sequencing can help reveal transmission chains and clusters of interest to aid outbreak investigations and surveillance efforts, as exemplified by the case studies above. With civet, academic researchers and public health scientists can easily run complex and robust phylogenetic analyses with a single command, contextualising sequences of interest in the large background dataset and visualising them alongside temporal, spatial and other epidemiological metadata in an interactive, distributable report. This frees users to place emphasis on interpreting the data and allows them to deliver information on a time-frame that is useful for public health responses.

Throughout the SARS-CoV-2 pandemic, civet has been primed for use investigating SARS-CoV-2 clinical outbreaks and running local surveillance on CLIMB-COVID [20] as part of the COG-UK project. Each day on CLIMB-COVID, researchers from around the UK upload the latest SARS-CoV-2 genome sequences and accompanying metadata. The read data undergo rigorous quality control and a data-processing and phylogenetics pipeline compiles and analyzes the resulting genomes in combination with the global dataset from GISAID (https://github.com/COG-UK/datapipe). This makes the latest SARS-CoV-2 genome data available to civet users on a daily basis. COG-UK data protection stipulates that data cannot be removed from CLIMB-COVID and often outbreak investigations involve sensitive, protected metadata. With civet, researchers can run analysis on CLIMB-COVID, distribute the report and keep their metadata protected. Civet has been popular and widely used within the framework of COG-UK, by academic researchers and scientists in public health agencies, for investigating SARS-CoV-2 clinical outbreaks and running local surveillance. A similar centralised server infrastructure could be set up for a national surveillance response or more local “locked down” compute environments [20] and civet could be easily implemented within this framework to aid outbreak investigations.

Civet can easily perform phylogenetic analysis on large datasets and provide reports for any countries with sequences to analyse. Default settings are configured for SARS-CoV-2, but civet is virus-agnostic and can be set up to run on other viruses of interest with an appropriate background dataset and reference sequence. Although civet is currently a command-line based tool, a clear extension to the software is to develop and provide a graphical user interface. This will enable users unfamiliar with the command line to run civet. We also plan to continue developing civet and adding extra features, including a country specific summary comparing counts of genomes sequenced over time with additional epidemiological data such as cases per country over time, which is already available on the Johns Hopkins University COVID-19 DataAPI [32]. This particular feature will help give appropriate context for countries with relatively low numbers of sequences as it is important to keep sequencing biases into account when inferring outbreak or transmission dynamics.

As the ability to rapidly sequence pathogens at scale has become less technically challenging, in part due to the availability of robust protocols such as those by the ARTIC Network [4], the amount of data that can be generated from a small laboratory with limited infrastructure has significantly increased. Arguably the greatest challenges now lay at trying to best utilise this data in an effective way to inform the response efforts, which hinges entirely on the ability to efficiently contextualise the data and provide an output that is interpretable by those less versed in the interpretation of phylogenetic trees. In this way, civet can help alleviate the analytical bottleneck that exists as a major issue for many public health labs and can maximise the value of genomic data.

## Supporting information

S1 Table Commands index. (DOCX)

S1 Fig Set categories for ‘updown-top-ranking’. (DOCX)

S2 Fig Impact of sequence quality on catchment accuracy. (DOCX)

S1 Text Gofasta tests. (DOCX)

S2 Text Supplementary author list. The COVID-19 Genomics UK (COG-UK) Consortium. COVID-19 impact project (Trinidad and Tobago Group).
(DOCX)
