# Supplementary material for: Genomics-informed outbreak investigations of SARS-CoV-2 using civet
Source: PLOS Glob Public Health. 2022 Dec 9;2(12):e0000704. doi: 10.1371/journal.pgph.0000704 (PMC10021969; doi:10.1371/journal.pgph.0000704)
Supplement: S1 Table — (DOCX) [file pgph.0000704.s001.docx]

**S1 Table** Commands index

|  | Case | Versions | Command |
| --- | --- | --- | --- |
| 1 | Hospital outbreak | civet v3.0  pangolin v3.1.10  pangoLEARN v2021-07-28  COG-UK data 2020-10-21 | civet  -i metadata.csv \  --timeline-dates sample_date \  -ds mode=enrich adm2=Edinburgh \  -ta HCW_status,ward,lineage,country \  --query-table-content name,lineage,source,catchment,\  sample_date,country,adm1,\  HCW_status,ward \ |
| 2 | Community surveillance | civet v3.0  pangolin v3.1.10  pangoLEARN v2021-07-28  COG-UK data 2020-10-21 | civet  --from-metadata N501Y=Y country=UK \  sample_date=2020-09-01:2020-10-21 \  --mutations S:N501Y \  --max-tree-size 1500 \  --max-queries 2000 \  --tree-annotations S:N501Y,lineage,country |
| 3 | National surveillance | civet v3.0  pangolin v3.1.10  GISAID data 2020-01-01 | civet  --from-metadata country=Trinidad_and_Tobago \  -bmcol country \  -rc 1,2,3,4,5,6,8 \  -bmdr 2019-12-01:2020-12-31 \  --snp-distance-up 5 \  --catchment-background-size 400 \ |

### 
