# Supplementary material for: Genomics-informed outbreak investigations of SARS-CoV-2 using civet
Source: PLOS Glob Public Health. 2022 Dec 9;2(12):e0000704. doi: 10.1371/journal.pgph.0000704 (PMC10021969; doi:10.1371/journal.pgph.0000704)
Supplement: S1 Fig — (DOCX) [file pgph.0000704.s002.docx]

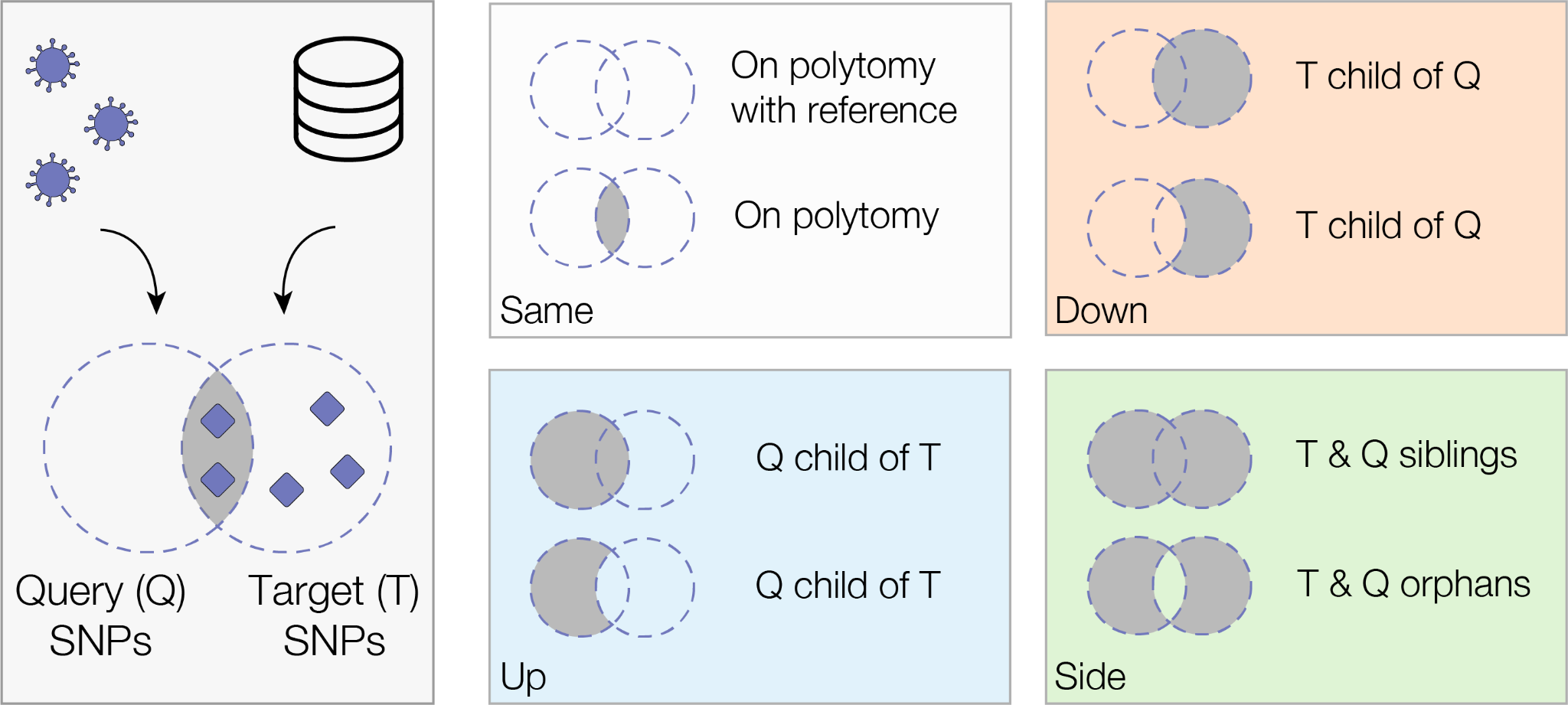


**S1 Fig**. Set categories for gofasta “updown-top-ranking”. Shaded regions in the Venn diagrams represent having at least one SNP in that category (either in Q, T or Q ∩ T).
