# Supplementary material for: Genomics-informed outbreak investigations of SARS-CoV-2 using civet
Source: PLOS Glob Public Health. 2022 Dec 9;2(12):e0000704. doi: 10.1371/journal.pgph.0000704 (PMC10021969; doi:10.1371/journal.pgph.0000704)
Supplement: S2 Fig — (DOCX) [file pgph.0000704.s003.docx]

*
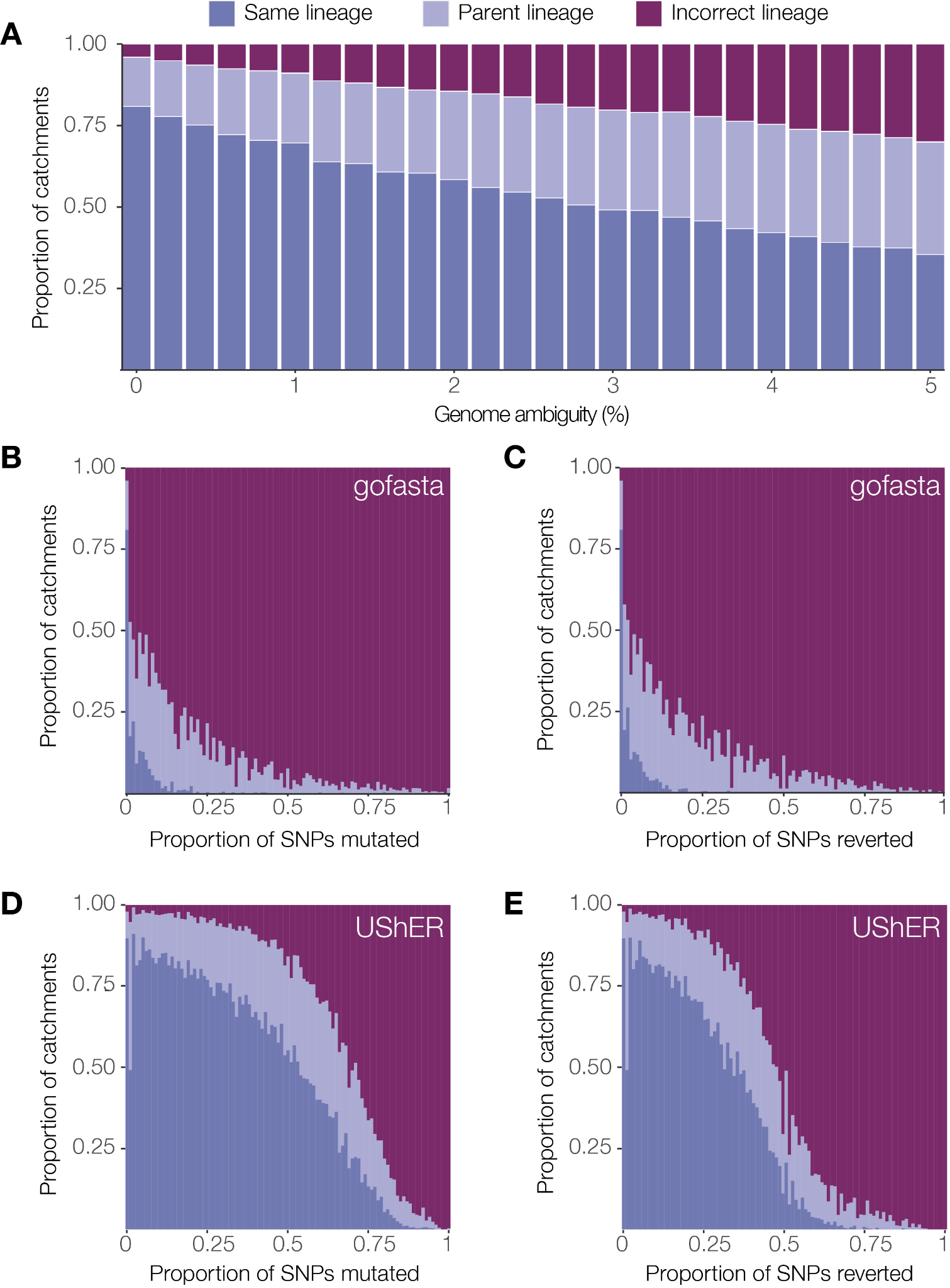
*

**S2 Fig.** A) Using Pango lineage as a proxy for appropriate catchment, we simulated increasing ambiguity randomly across the genome for a representative genome for each Pango lineage. As ambiguity increased, we assessed whether the correct lineage, a parental lineage or an inappropriate lineage was found. B) In a more targeted approach, we specifically mutated SNPs of a given query sequence to see how gofasta behaves in response to contradictory mutations at relevant sites, rather than just random mutations. As expected, missing the critical SNPs very quickly leads to incorrect lineage and catchment selection. A proportion of sequences get placed more root-ward within the parent diversity when missing crucial defining SNPs but once many of the defining SNPs are missing from a given sequence, it becomes impossible to correctly place the sequence. C) In a similar approach, we simulated reversions within the dataset by removing increasing proportions of the SNPs of a given query sequence. Without this SNP information it quickly becomes difficult to correctly place the sequence within the appropriate catchment. As a comparison, panels D and E show the lineage assignment results using the UShER mode of pangolin for the same test datasets used in panels B and C. Although this is not an exact measure of ‘correct catchment’ we believe correct lineage is an appropriate proxy for this. We see UShER is more robust to ambiguous data than gofasta for placement within the downsampled lineage tree used within pangolin.
