# Supplementary material for: Genomics-informed outbreak investigations of SARS-CoV-2 using civet
Source: PLOS Glob Public Health. 2022 Dec 9;2(12):e0000704. doi: 10.1371/journal.pgph.0000704 (PMC10021969; doi:10.1371/journal.pgph.0000704)
Supplement: S1 Text — (DOCX) [file pgph.0000704.s004.docx]

**S1 Text**

*Gofasta tests*

Missing data has been a big issue throughout the pandemic and there is a range in genome quality on public repositories and what is generated in the lab. In civet, we use gofasta, a tool that uses a novel approach to inferring related sequences in a tree-independent manner. In order to assess its use as part of the civet tool, a series of tests were performed on gofasta. Gofasta (v1.0.0) was tested using SARS-CoV-2 sequences sourced from GISAID on 2022-04-13. We downloaded the entire GISAID database and removed the genomes we were querying – i.e. (designated sequences sequences (<https://github.com/cov-lineages/pango-designation/blob/master/lineages.csv>; last accessed 15 June 2022) – so that we are not just matching the query sequence within the background data. The aligned fasta file of 9,316,976 SARS-CoV-2 sequences was transformed into a gofasta list file using the -list function. The representative of each lineage with the lowest number of ambiguous sites was removed from the background dataset and used to create the base query set (n = 1720), giving a high-quality set representing each different Pango lineage. All remaining designated sequences formed the search dataset (n = 1,530,767). For each test on gofasta updown topranking, --dist-all was set to 1 and the --dist-push flag included, allowing the tool to extend the SNP distance in the event of an empty bin.

We used the lineage composition of the catchment found by gofasta as a measure of gofasta finding the appropriate catchment. If the majority of sequences within the catchment are the correct lineage, we report the lineage is the same. If the majority of sequences within the found catchment belong to the nearest ancestral lineage (which in cases of missing SNPs is what we’d expect), we report parent lineage. Finally, if a lineage other than the correct or parent lineage is the majority lineage found in the catchment, we report an incorrect lineage. We believe this categorisation is very conservative but does reflect the catchment accuracy of gofasta.

While initial tests were performed on the designated query set, selected to minimise ambiguities, this does not reflect the majority of SARS-CoV-2 sequences. In order to assess the performance of gofasta on real data, a series of tests were designed to simulate common errors, such as incorrect reversions to the reference genome or missing data.

The first test simulated random ambiguities (N bases) across each representative genome with increasing levels of ambiguities. We also ran simulations targeting specifically the mutations within the query sequences. We tested simulated query genomes with conflicting mutations at these sites as well as genomes missing the appropriate SNPs. As gofasta uses the SNP information to place sequences, we anticipate that targeting these SNPs will very quickly lead to a decline in catchment accuracy.

The simulated errors in the SNP set were introduced to the original query list file according to two patterns. These included 1) novel mutations, where the new base is neither the ancestral nor the pre-existing derived allele and 2) reversions to the ancestral allele, where the SNP is removed from the query set (Supplementary Figure 2C-D). In order to test the effect of missing data on gofasta performance, a series of simulated query datasets were created. Ambiguities were introduced at random throughout the sample genomes in steps of 0.2%, from 0 to 5% (Supplementary Figure 2B).

The effect of search database size on the tool’s accuracy in lineage assignment was also investigated. Alternate databases were created with increasing numbers of representatives of each lineage, starting at 1 and increasing in intervals of 5 until 100, where sequences were available. gofasta was then run as described above with the default query set (n = 1720) for each search database.

### Supplementary Results

*Impact of sequence quality on catchment accuracy*

During the SARS-CoV-2 pandemic, global genomics capacity has massively improved. However, as is the case with many sharing platforms, the global SARS-CoV-2 database has a wide range of genome quality, with genomes often having missing data, amplicon dropouts and incorrect reversions to reference. As such, all downstream inference of SARS-CoV-2 has the potential to be impacted by the quality of the background database provided as well as the quality of query sequences. By using Pango lineage as a good proxy for correct catchment, we assessed how gofasta behaves in response to increasing database size, and simulated ambiguity and diversity (Supplementary Figure 2).

In Supplementary Figure 2A-C, we investigate how gofasta’s updown topranking method behaves in response to varying quality data by simulating ambiguities and reversions. Making the assumption that the sequences within the background data have all been assigned a correct Pango lineage, we use finding the correct Pango lineage in the background data as a proxy for appropriate catchment found. In panel B, we simulated random ambiguity across the genome and saw a decline in accuracy as the query genomes have higher levels of missing data.

From a practical point of view, random ambiguities could have very little impact on the ability of gofasta to appropriately place a given query sequence if the small number of important sites are left unmutated. For a given genome in a lineage with 5 defining SNPs, all 5 SNPs could be missing by chance with an otherwise very complete genome (>99%). If these 5 critical sites are missing, the average ambiguity percent for the genome as a whole is irrelevant. As such, we next simulated targeted ambiguities at defining sites. In addition to ambiguities, reversion mutations are common in the global SARS-CoV-2 dataset, often present due to issues in upstream bioinformatic processing of the read data to generate the genome sequences. The simulations of targeted ambiguities are applicable to assment of reversions as they would appear the same within the gofasta SNP file. As gofasta uses the SNP information to place sequences, we anticipate that targeting these SNPs will very quickly lead to a decline in catchment accuracy.

As expected, in Supplementary Figure 2C our results show that catchment accuracy declines rapidly with reversion mutations. Reversions will make descendants of a sequence look like they have some other relationship with their ancestors (depending on how many other mutations have accumulated). Because of this they will be categorised into the wrong bin, but they will still be in some bin at an appropriate distance. For our tests however, the assessment of accurate catchment was very conservative at assessing catchment accuracy and if placed in a ‘side’ bin this would get classified as inaccurate. In any case, at the genetic distances and timescales relevant for outbreak investigations and short term surveillance, reversion mutations are most likely to be due to bioinformatic errors or recombination events.

Overall, we see in our tests that targeted mutation and targeted ambiguities very quickly impact the accuracy of gofasta’s placement. We observe that simulating targeted conflicting mutations and targeted removal of SNPs in the query sequence, which corresponds to missing data or reversions in the data, leads to a sharp decline in the ability of gofasta to recover an appropriate catchment. As this method appears sensitive to missing data, we recommend that the background dataset and query sequences should be of the highest quality as possible. We performed the same tests on UShER and, similar to our assessment of gofasta, used it’s lineage assignment as a proxy for accurate catchment retrieval (Supplementary Figure 2 D-E) and found it to be less sensitive to missing data or incorrect data than gofasta. Users may wish to independently validate the catchments using hgPhyloplace.
